# P-1834. An Enteric Bacterial Infection Disrupts Brain Homeostasis and Induces Chronic Neuroinflammation by Breaching the Gut-Brain Axis in 3xTg-AD Transgenic Mice

**DOI:** 10.1093/ofid/ofae631.1997

**Published:** 2025-01-29

**Authors:** Gwoncheol Park, Saurabh Kadyan, Nathaniel Hochuli, Ravinder Nagpal

**Affiliations:** Florida State University, Tallahassee, Florida; Florida State University, Tallahassee, Florida; Florida State University, Tallahassee, Florida; Florida State University, Tallahassee, Florida

## Abstract

**Background:**

In previous studies, we found that an enteric infection caused by *Klebsiella pneumoniae* (*Kpn*) not only disturbs the balance of gut microbiome but also translocates to the brain, leading to inflammation and impairments in neurobehavior.

**Methods:**

Triple-transgenic (3xTg-AD) mice were split into three categories: those infected with *Kpn* (Kpn group), a subset of *Kpn* mice treated with antibiotics to induce dysbiosis (Kpn+Ab group), and a control group receiving no treatment (TgCt group). Metabolomic and inflammation profiles of gut, serum, and brain samples collected six weeks after infection were evaluated using NMR-based metabolomic analysis and transcriptomics analysis conducted with the NanoString nCounter system.

**Results:**

Significant shifts were observed in gut metabolites, while serum metabolites showed minor changes, particularly with an upward trend in branched-chain amino acids in *Kpn*-treated mice. In the gut, an inflammatory response was evident, characterized by the increased expression of inflammatory genes such as *Cd14, Ptgs2,* and *Il6st*. Transcriptomic analysis of the brain revealed disrupted homeostasis, with heightened neuropathological processes, including oxidative stress and cytokines, and reduced structural and anti-pathological functions, particularly notable in the Kpn+Ab group. Specifically, genes associated with inflammation and neurodegeneration, such as *Il1r1* and *Ptgs2*, were upregulated in the Kpn+Ab group, suggesting a chronic inflammatory state potentially worsening neuropathology. Indeed, elevated levels of total tau were observed in the brains of Kpn+Ab mice. Furthermore, dysregulation in redox signaling linked to the malate-aspartate shuttle was evident, as indicated by increased levels of aspartate and N-acetylaspartate alongside decreased serine concentrations.

**Conclusion:**

The results indicate that *Kpn* enteric infection leads to persistent inflammation in both the gut and the brain, disrupting essential pathways for brain homeostasis. This disruption may contribute to the buildup of AD pathologies and impairments in neurobehavioral function.

**Disclosures:**

**All Authors**: No reported disclosures

